# Effectiveness of Ring Vaccination as Control Strategy for Ebola Virus Disease

**DOI:** 10.3201/eid2201.151410

**Published:** 2016-01

**Authors:** Adam J. Kucharski, Rosalind M. Eggo, Conall H. Watson, Anton Camacho, Sebastian Funk, W. John Edmunds

**Affiliations:** London School of Hygiene and Tropical Medicine, London, UK

**Keywords:** Ebola virus disease, EVD, Ebola virus, viruses, ring vaccination, vaccination, transmission chains, mathematical model, Guinea, West Africa

## Abstract

Using an Ebola virus disease transmission model, we found that addition of ring vaccination at the outset of the West Africa epidemic might not have led to containment of this disease. However, in later stages of the epidemic or in outbreaks with less intense transmission or more effective control, this strategy could help eliminate the disease.

During 2014–2015, trials of candidate vaccines for Ebola virus disease (EVD) were fast tracked in response to the unprecedented EVD epidemic in West Africa (*1*). In March 2015, a phase 3 ring vaccination trial of a recombinant vesicular stomatitis virus–Zaire Ebola virus vaccine began in Guinea (*2*). Interim trial results suggested that the vaccine could have a high level of efficacy in humans (*3*). Ring vaccination has also been used for disease control, notably in the final stages of the smallpox eradication program (*4*). Furthermore, a recent modeling study calibrated by using population-level EVD data from Sierra Leone and Liberia (*5*) suggested that ring vaccination could supplement case isolation and contact tracing in reducing transmission. However, it remains unclear whether prompt ring vaccination, as opposed to large-scale mass vaccination, could have contained the EVD epidemic in West Africa, and under what circumstances it could be effective in controlling future outbreaks.

## The Study

**Figure 1:**
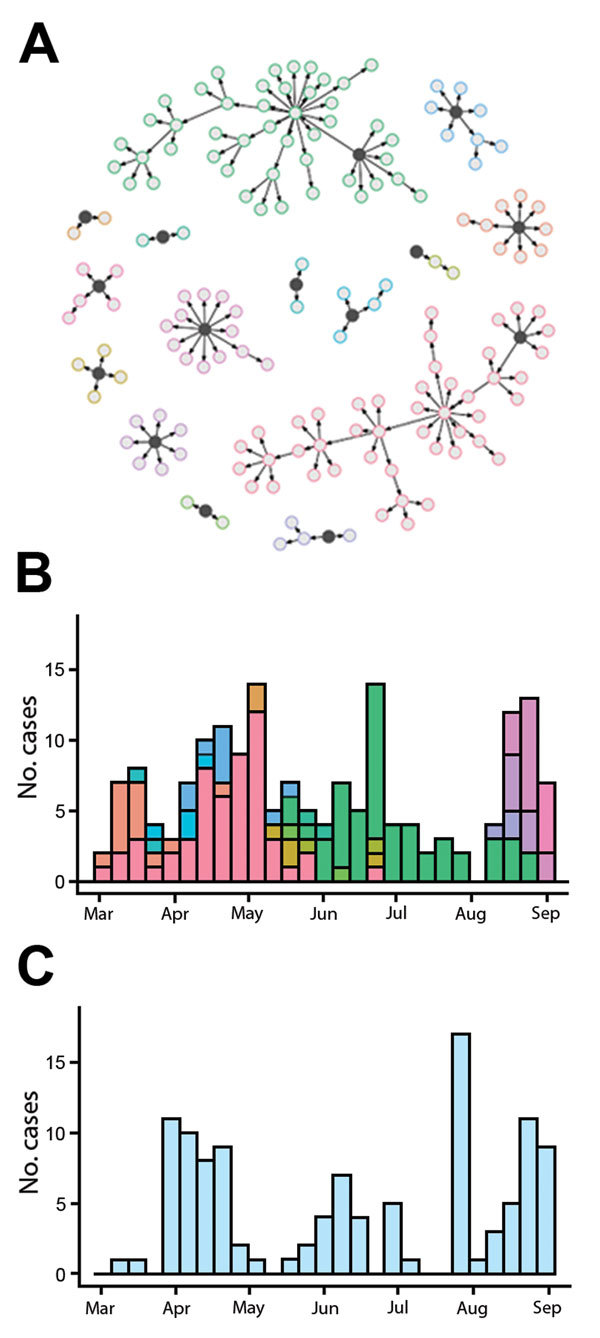
Outbreak dynamics in a model of transmission of Ebola virus disease. A) Chains of transmission generated in a simulated outbreak starting with 2 infected persons on March 1, 2014. Black circles indicate the index case within each cluster, and arrows indicate routes of transmission. Within each cluster, we assumed that there was a 15% probability that a secondary case would be missed and would instead seed a new cluster (these missed links are not shown). B) New cases per week, by date of symptom onset, for the chains of transmission shown in panel A. Colors of clusters in panel A match colors of bars in panel B. C) Observed weekly confirmed and probable cases reported in Conakry Prefecture, Guinea, during March–September 2014. Data were obtained from the Guinea Ministry of Health and World Health Organization Situation Reports (*11*).

We developed a stochastic model of EVD transmission (Technical Appendix) using individual-level transmission data from Guinea to inform our model structure. Transmission chains during March–August 2014 suggest substantial variation in the number of secondary cases generated (*6**,**7*). In particular, index cases, defined as those that could not be linked to an already known transmission chain, had a reproduction number of *R_m_* = 7, where *m* indicates missed and *R* denotes the average number of secondary cases generated, whereas cases within known transmission chains (*w*) had a reproduction number of *R_w_* = 0.66 (Technical Appendix, Figure 1).

**Figure 2:**
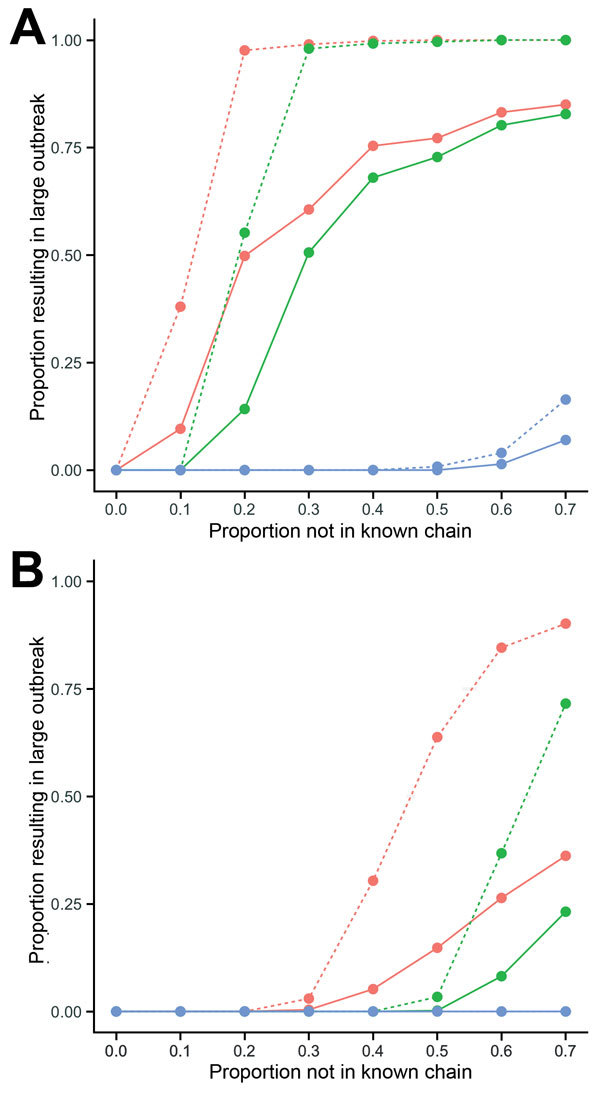
Effectiveness of vaccination strategies for Ebola virus disease under different transmission scenarios. A) Proportion of simulations that led to a large outbreak (defined as >500 clusters) in the early 2014 Guinea transmission scenario. Red lines indicate no vaccination, green lines indicate ring vaccination, blue lines indicate mass vaccination; solid lines indicate outbreaks that started with 1 index case, and dashed lines indicate outbreaks that started with 5 index cases. We simulated 1,000 outbreaks and calculated the proportion that resulted in >500 clusters. When the space between the red and green lines is large, the model suggests that ring vaccination would provide substantial additional value over standard public health control measures alone. B) Proportion of simulations that led to a large outbreak in partial control scenario.

In the model, transmission followed a branching process (*8*), and secondary cases were generated from a negative binomial distribution to include potential for superspreading events (*6**,**9*). Each cluster started with an index case, which generated an average of *R_m_* = 7 secondary cases. Many EVD cases reported in Guinea were not part of already known transmission chains (Technical Appendix, Figure 2). We therefore assumed there was a probability ρ that a secondary case would missed and go on to seed an independent transmission cluster as an index case with *R_m_* = 7. Otherwise, the case would remain within the known chain of transmission (with probability 1 – ρ); these cases would then generate an average of *R_w_* = 0.66 secondary cases. The simulated outbreaks ended when, by chance, no secondary cases were generated by active cases. Distributions of incubation period, duration of infectiousness, and time to reporting were obtained from reported values for Guinea in 2014 (*10*). Model simulations produced similar patterns to those observed in 2014 (Figure 1). When half of the cases were missed, the overall reproduction number, defined as the mean number of secondary cases generated across all infectious persons, was ≈1.5, which was similar to values observed in early 2014 in West Africa (*11*) and in the initial stages of other outbreaks (Technical Appendix Table 1).

We simulated ring vaccination by using a protocol similar to that used in Guinea trial (*3*). We defined a ring as all persons who could potentially form part of the known chain of transmission (i.e., traceable contacts of infected persons within a transmission cluster and their contacts). Once the index case was reported, we assumed it took 2 days to vaccinate a ring and that protective immunity developed 7 days after vaccination. In the model, we assumed that vaccine efficacy was 80% and that 70% of the ring received vaccination (Technical Appendix). The reproduction number within a ring was therefore reduced by a factor of 1 – (0.8 × 0.7) = 0.44 once the vaccine became effective (Technical Appendix Figure 3). To estimate the effect of ring vaccination, we simulated multiple outbreaks and calculated the proportion of these outbreaks that became large (i.e., >500 clusters). We found that if more than a few cases were missed, large outbreaks could occur under ring vaccination (Figure 2, panel A). This event could occur because missed cases, which had a higher reproduction number, would not be inside the ring when vaccination was introduced. Although ring vaccination failed to contain the outbreak in this scenario, it still reduced disease transmission (Technical Appendix Figure 4). We also considered the effect of preemptive mass vaccination, which reduced the reproduction number for all cases by a factor of 0.44, regardless of whether cases were in the cluster or missed. This strategy was more effective in containing outbreaks, even if many cases were missed (Figure 2, panel A). Similar qualitative patterns were observed when vaccine efficacy was 95% (online Technical Appendix Figure 5).

In the later stages of the EVD epidemic in West Africa, behavior changes and improved control measures led to transmission from burials and in hospital settings than in early 2014 (*12*). Similar reductions were observed in other Ebola outbreaks (e.g., in 1976 in Yambuku, Zaire) (*13*). We therefore also explored a partial control scenario. We omitted index cases in the 2014 Guinea transmission chains that were involved in funeral or hospital transmission, which resulted in *R_m_* = 2.5 for missed cases (Technical Appendix Figure 6). We also assumed a shorter duration of infectiousness and time to reporting on the basis of data for 2015 (*3**,**10*) (Technical Appendix Table 2).

In this partial control scenario, outbreaks could be controlled with ring vaccination, even if 40% of cases were missed (Figure 2, panel B). Our results suggest that ring vaccination could substantially reduce the potential size and duration of outbreaks if other control measures are also in place (Table). We also estimated how many vaccine doses would be required for ring vaccination (Technical Appendix); in the partial control scenario, several thousand doses might be needed (Technical Appendix Table 3). We could not estimate doses required for mass vaccination, and thus could not perform an economic analysis of different strategies, because this would depend on the potential for long-distance transmission events and populations in different areas. However, implementing mass vaccination for even a single district in West Africa could require >100,000 doses.

**Table:** Estimated total cases and outbreak duration in partial control scenario with 5 index cases initially by using the model of Ebola virus transmission*

Probability of case missed	No vaccination	Ring vaccination	Mass vaccination
Median no. cases (95% CI)			
10%	42 (14–235)	30 (13–79)	13 (7–60)
20%	63 (15–551)	39 (14–131)	13 (7–57)
30%	104 (17–2,660)	53 (15–229)	13 (6–48)
40%	296 (20–2,410)	78 (18–452)	13 (6–46)
Duration of outbreak, d (95% CI)			
10%	87 (28–278)	62 (26–145)	41 (12–139)
20%	123 (33–480)	83 (31–214)	43 (11–149)
30%	185 (43–1,020)	110 (36–319)	47 (11–142)
40%	364 (51–1,150)	149 (45–486)	47 (9–147)

*Model assumes 80% vaccine efficacy.

Our analysis has some limitations. In the early 2014 transmission scenario, we assumed that missed cases had a much higher reproduction number than cases within clusters. However, if an effective vaccine became available, persons at risk might be more likely to engage with public health efforts. The high reproduction number for index cases might also be caused in part by ascertainment bias: cases that generate many secondary infections are more likely to be designated as index cases. We also assumed that mass vaccination would target 70% of the population at random; in practice, there could be clustering effects. Furthermore, we assumed that chains of transmission were independent and that the reproduction number remained unchanged over time. In reality, missed cases might have shared contacts and behavior might change during outbreak, which could reduce transmission. Our estimates are therefore likely to represent a reasonable worst-case scenario.

## Conclusions

Ring vaccination enhances standard public health measures of contact tracing, isolation, and community engagement (*14*) and could be effective when such measures are in place. However, if standard measures are not working because many cases are not in known transmission chains, as in West Africa in early 2014, ring vaccination might be insufficient to contain the outbreak. If an EVD vaccine is shown to be efficacious, our results suggest that mass vaccination, or hybrid strategies involving mass and ring vaccinations, might need to be considered alongside ring vaccination when planning for future outbreaks.

**Technical Appendix.** Additional information on effectiveness of ring vaccination as control strategy for Ebola virus disease.
