# Supplementary material for: Effectiveness of Ring Vaccination as Control Strategy for Ebola Virus Disease
Source: Emerg Infect Dis. 2016 Jan;22(1):105–8. doi: 10.3201/eid2201.151410 (PMC4696719; doi:10.3201/eid2201.151410)
Supplement: Supplementary file 1 — Technical Appendix. Additional information on effectiveness of ring vaccination as control strategy for Ebola virus disease. [file 15-1410-Techapp-s1.pdf]

Article DOI: http://dx.doi.org/10.3201/eid2201.151410

Effectiveness of Ring Vaccination as Control Strategy for Ebola Virus Disease

Technical Appendix

Transmission Model

In the model, transmission followed a branching process, and secondary cases were generated from a negative binomial distribution (*1*,*2*). We estimated the reproduction number for missed cases, R_m, and for cases within a cluster, R_w, by fitting negative binomial distributions (*3*) to data on chains of transmission in Conakry, Guinea, during March–August 2014 (*4*). The distribution of secondary cases in this dataset was enumerated by Althaus (*1*). Our maximum likelihood estimates were $R_m = 7$ and $R_w = 0.66$ (Technical Appendix Figure 1, panels A, B). We also obtained estimates of the dispersion parameters (Technical Appendix Table 1), which suggested that transmission from cases within clusters was highly overdispersed (k = 0.19). This estimate indicated that although R_w was small, there was still potential for superspreading events within each cluster. As a validation, we also analyzed published data on a transmission chain in Liberia, which occurred during December 2014–March 2015 (*14*). The results were similar: the index case had $R_m = 5$ and the secondary cases generated by nonindex cases had $R_w = 0.76$ and $k = 0.82$ (Technical Appendix Figure 1, panel C).

In our transmission model, each cluster started with an index case, which generated secondary cases from the fitted negative binomial distribution with average R_m. We assumed there was a probability $1 - \rho$ that each secondary case would remain within the known chain of transmission; these cases would then generate an average of R_w secondary cases. If a case was missed (with probability ρ), it went on to seed a new cluster as an index case with reproduction number R_m. The simulated outbreaks came to an end when no secondary cases were generated by the currently active cases.

In the Conakry transmission scenario, we assumed that incubation period, duration of infectiousness, and time from onset to notification were gamma distributed, and had a mean and

SD as in the World Health organization patient database for Guinea (*5*). The duration of infectiousness was equal to the time from onset to death (6.4 days).

In the partial control scenario, we assumed that this was equal to the time from onset to hospitalization (5.3 days) because of the increased proportion of cases that would have been isolated. Note that these parameters do not affect the number of secondary cases generated by an infectious person (which is specified by R_m and R_w), but do affect the duration of the outbreak. The incubation period remained unchanged, but the average time from onset to reporting decreased 3.6 days, as reported in the 2015 Guinea ring vaccination trial (*15*). The proportion of cases in known chains during this period is shown in Technical Appendix Figure 2. The model was implemented in R version 3.1.3 (*17*).

Comparison of Overall Reproduction Numbers in Simulated and Real Outbreaks

To compare our 2 simulated scenarios with transmission dynamics in real Ebola virus disease outbreaks, we calculated the overall reproduction number in outbreak simulations. This number was defined as the mean number of secondary cases generated across all infectious persons. For example, in the transmission chain shown in Figure 1, the overall reproduction number R = 0.88. The mean overall reproduction number for the scenarios shown in Figure 2 are shown in Technical Appendix Figure 4. As the reproduction number approaches the critical value of 1 in Technical Appendix Figure 4, on average transmission will become self-sustaining in the model, and thus the probability of observing a large outbreak increases substantially in Figure 2.

When 20%–70% of cases are missed in the model, the overall reproduction number for the Conakry scenario ranges between 1 and 2 if outbreaks start with a single initial case in the absence of vaccination (Technical Appendix Figure 4, panel A). This finding is consistent with estimates of the reproduction number for the West Africa epidemic in early 2014, and with estimates of the community reproduction number from the early period of other outbreaks (Technical Appendix Table 1). Detailed transmission chain data are not available for historical outbreaks, and thus scenarios we consider might not exhibit the same individual variation in secondary transmission (as specified by the dispersion parameter *k*). However, the similarity in overall reproduction number suggests that the average transmission dynamics are comparable.

However, the crucial difference between pre-2013 outbreaks and the situation in West Africa during 2013–2015 is that previously the overall reproduction number decreased substantially within weeks rather than over months or years. Thus, our partial control scenario, in which the mean overall reproduction number is >1 even without vaccination (Technical Appendix Figure 4, panel B) is likely to be more representative of the transmission patterns typically faced during earlier EVD outbreaks.

Ring Vaccination

During the 2015 Guinea recombinant vesicular stomatitis virus–Zaire Ebola virus vaccine trial, ring vaccination was implemented by vaccinating all named contacts of confirmed index case-patients, as well as other persons who would have been at risk because of their connections to the case-patient; for example, the village of the case-patient, or household contacts of a named high-risk contact that lived far from the case-patient (*15*). In the model, we therefore assumed that a randomly sampled proportion of all persons who could form part of the transmission cluster (i.e., were not missed) would be vaccinated.

Once the index case was reported, which depended on the time from onset to reporting, it took 2 days for persons to be vaccinated. We assumed that the vaccine became effective after an additional 7 days because interim results suggested that no infections were observed in vaccinated persons in the Guinea ring vaccination trial after $\geq$6 days (*15*). We also assumed an efficacy of 80%, which is toward the lower end of the range given in the interim results from the Guinea trial (*15*). As a sensitivity analysis, we repeated our simulations assuming an efficacy of 95% (Technical Appendix Figure 5).

In the trial, 5,415 (71%) of 7,651 contacts were >18 years of age and not pregnant or breastfeeding, and thus were eligible for vaccination. Overall, 52% were eligible and consented. Because vaccination will in the future likely be expanded to younger age groups, in the model, we therefore assumed that a uniformly random sample of 70% of the cluster received the vaccine. The reproduction number within a cluster was therefore reduced by a factor (1 – 0.8 × 0.7) = 0.44 once the vaccine became effective. Thus, the reproduction number was equal to R_w before vaccine becomes effective and $R_v = 0.44\ R_w$ afterwards.

We also estimated how many vaccine doses would be required to conduct out such ring vaccination. On the basis of the number of persons vaccinated in each cluster in the Guinea

recombinant vesicular stomatitis virus–Zaire Ebola virus vaccine trial (*15*), we specified the vaccination ring size to be normally distributed with a mean size of 80 persons and an SD of 20, with each new transmission cluster requiring this number of persons to be vaccinated. In the partial control scenario, several thousand doses may therefore be required to implement ring vaccination (Technical Appendix Tables 2, 3).

Mass Vaccination

To introduce mass vaccination in the model, we assumed that 70% of all persons in at-risk areas were vaccinated; efficacy = 80% (sensitivity analysis with 95% efficacy is shown in Technical Appendix Figure 4). This assumption suggests the proportion of the population who were susceptible to infection was reduced by a factor of 0.44, regardless of whether they were in the cluster or missed. In our branching process model, this assumption was implemented by reducing R_m and R_w by a factor of 0.44. When there is 1 initial case, our framework is equivalent to the assumption that mass vaccination was performed preemptively before that specific outbreak began. When there are several initial cases, we are making the assumption that large-scale mass vaccination was conducted in the short period between the first case being reported and the start point of the simulation, by which multiple initial cases were infected.

References

1. Althaus CL. Ebola superspreading. Lancet Infect Dis. 2015;15:507–8. PubMed http://dx.doi.org/10.1016/S1473-3099(15)70135-0

2. Lloyd-Smith JO, Schreiber SJ, Kopp PE, Getz WM. Superspreading and the effect of individual variation on disease emergence. Nature. 2005;438:355–9. PubMed http://dx.doi.org/10.1038/nature04153

3. Delignette-Muller M, Dutang C, Pouillot R, Denis JB. Fitdistrplus package; 2015 [cited 2015 Oct 22]. https://cran.r-project.org/package=fitdistrplus

4. Faye O, Böelle PY, Heleze E, Faye O, Loucoubar C, Magassouba N, et al. Chains of transmission and control of Ebola virus disease in Conakry, Guinea, in 2014: an observational study. Lancet Infect Dis. 2015;15:320–6. PubMed http://dx.doi.org/10.1016/S1473-3099(15)71075-8

5. WHO Ebola Response Team. Ebola virus disease in West Africa—the first 9 months of the epidemic and forward projections. N Engl J Med. 2014;371:1481–95. PubMed http://dx.doi.org/10.1056/NEJMoa1411100

6. Camacho A, Kucharski AJ, Funk S, Breban J, Piot P, Edmunds WJ. Potential for large outbreaks of Ebola virus disease. Epidemics. 2014;9:70–8. PubMed http://dx.doi.org/10.1016/j.epidem.2014.09.003

7. Chowell G, Hengartner NW, Castillo-Chavez C, Fenimore PW, Hyman JM. The basic reproductive number of Ebola and the effects of public health measures: the cases of Congo and Uganda. J Theor Biol. 2004;229:119–26. PubMed http://dx.doi.org/10.1016/j.jtbi.2004.03.006

8. Ferrari MJ, Bjørnstad ON, Dobson AP. Estimation and inference of R0 of an infectious pathogen by a removal method. Math Biosci. 2005;198:14–26. PubMed http://dx.doi.org/10.1016/j.mbs.2005.08.002

9. Legrand J, Grais RF, Boelle PY, Valleron AJ, Flahault A. Understanding the dynamics of Ebola epidemics. Epidemiol Infect. 2007;135:610–21. PubMed http://dx.doi.org/10.1017/S0950268806007217

10. Althaus CL. Estimating the reproduction number of Ebola virus (EBOV) during the 2014 outbreak in West Africa, March to August 2014. PLoS Curr. 2014;6:pii: ecurrents.outbreaks.91afb5e0f279e7f29e7056095255b288.

11. Nishiura H, Chowell G. Early transmission dynamics of Ebola virus disease (EVD), West Africa, March to August 2014. Euro Surveill. 2014;19:pii: 20894. PubMed

12. Centre for the Mathematical Modelling of Infectious Diseases. Visualisation and projections of the Ebola outbreak in West Africa [cited 2015 Oct 21]. http://cmmid.lshtm.ac.uk/ebola/

13. Camacho A, Kucharski AJ, Aki-Sawyer Y, White MA, Flasche S, Baguelin M, et al. Temporal changes in Ebola transmission in Sierra Leone and implications for control requirements: a real-time modelling study. PLoS Curr. 2015;7:pii: ecurrents.outbreaks.406ae55e83ec0b5193e30856b9235ed2.

14. Nyenswah T, Fallah M, Sieh S, Kollie K, Badio M, Gray A, et al. Controlling the last known cluster of Ebola virus disease—Liberia, January–February 2015. MMWR Morb Mortal Wkly Rep. 2015;64:500–4. PubMed

15. Henao-Restrepo AM, Longini I Jr, Egger M, Dean NE, Edmunds WJ, Camacho A, et al. Efficacy and effectiveness of an rVSV-vectored vaccine expressing Ebola surface glycoprotein: interim results from the Guinea ring vaccination cluster-randomised trial. Lancet. 2015;386:857–66. PubMed http://dx.doi.org/10.1016/S0140-6736(15)61117-5

16. World Health Organization. Weekly epidemiological situation. Ebola outbreak in Guinea: week 20, 2015 [in French] [cited 2015 Oct 21]. http://guinea-ebov.github.io/sitreps.html

17. R Core Team. R: a language and environment for statistical computing. Vienna, Austria; 2014 [cited 2015 Oct 21]. http://www.R-project.org/

Technical Appendix Table 1. Estimates of overall community reproduction number in historical Ebola outbreaks and in early stages of the 2013–20515 Ebola virus disease epidemic in West Africa

Location	Date	Reproduction no.	Reference
Yambuku, Zaire	1976	1.34	(*6*)
Kikwit, Zaire	1995	1.83	(*7*)
		3.65	(*8*)
		2.7	(*9*)
Uganda	2000–2001	1.34	(*7*)
		1.79	(*8*)
		2.7	(*9*)
Guinea	2014 Mar–Aug	1.5	(*10*)
	2014 Mar–Aug	≈1	(*11*)
	2014 Jul–Sep	1.71	(*5*)
	2014 Jul–Oct	1.2–1.7	(*12*)
Sierra Leone	2014 Mar–Aug	2.5	(*10*)
	2014 Jun–Jul	1.4	(*11*)
	2014 Jul–Sep	2.02	(*5*)
	2014 Jul–Oct	1.3–1.5	(*12,13*)
Liberia	2014 Mar–Aug	1.59	(*10*)
	2014 Jun–Jul	1.7	(*11*)
	2014 Jul–Sep	1.83	(*5*)

Technical Appendix Table 2. Parameters used in the model for the 2 transmission scenarios for Ebola virus disease*

Parameter	Guinea, early 2014	Partial control
Reproduction no. for index cases, R_m	7 (k = 1.6)	2.5 (k = ∞)
Reproduction no. for secondary cases within cluster, R_w	0.66 (k = 0.19)	0.66 (k = 0.19)
Incubation period, d, mean ± SD	9.1 ± 7.3	9.1 ± 7.3
Time from onset to reporting, d, mean ± SD	7.5 ± 10.4	3.9 ± 2.6
Duration of infectiousness, d, mean ± SD	6.4 ± 5.3	5.3 ± 4.3
Proportion of persons within a cluster who are vaccinated, %	70	70
Vaccine efficacy, %	80–95	80–95
Vaccination ring size, mean ± SD	80 ± 20	80 ± 20

*The fitted dispersion parameter *k* for the negative binomial distributions is given along with reproduction numbers. We assumed that incubation period, time from onset to reporting, and duration of infectiousness followed a gamma distribution.

Technical Appendix Table 3. Estimate doses required for elimination of Ebola virus disease with a ring vaccination strategy in the partial control scenario*

Probability of case missed	No. doses required (95% CI)
10%	382 (175–822)
20%	533 (216–1,440)
30%	774 (265–2,900)
40%	1,220 (329–6,760)

*Outbreaks started with 5 index cases and 80% vaccine efficacy.

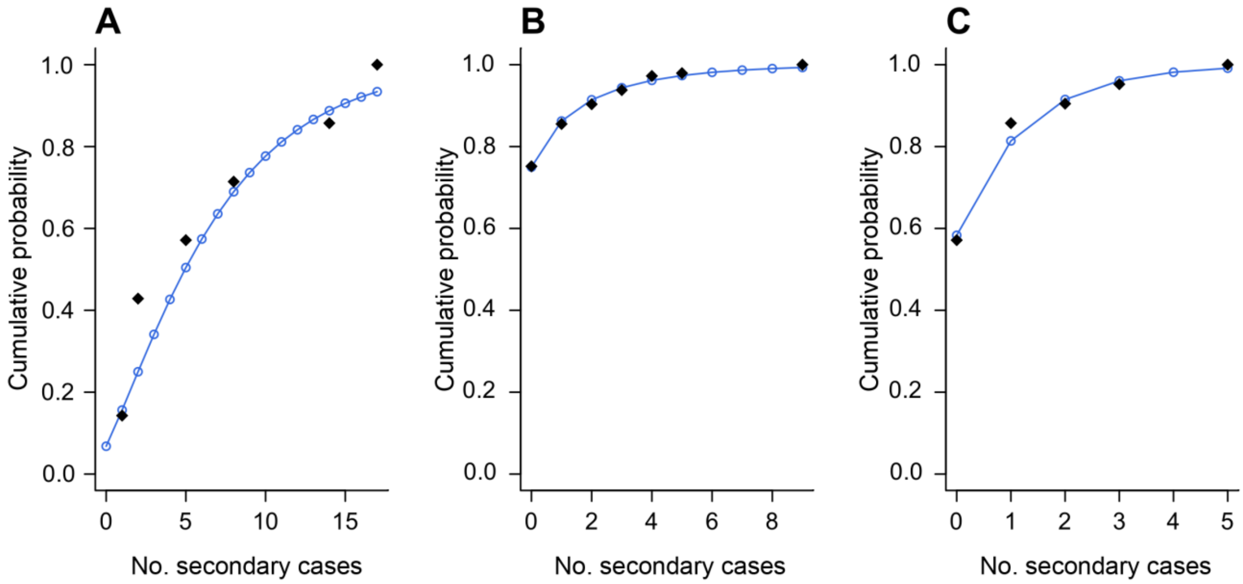

Technical Appendix Figure 1. Fitted distributions of reproduction numbers for effectiveness of ring vaccination as control strategy for Ebola virus disease. A) Cumulative distribution of secondary cases generated by index cases in transmission chains in Conakry, Guinea, March–August 2014 (*4*). Blue line indicates fitted negative binomial distribution, blue circles indicate values of the distribution at integer intervals, and black diamonds indicate data. B) Secondary cases generated by cases within clusters in Conakry. C) Secondary cases generated by nonindex cases in Liberia cluster.

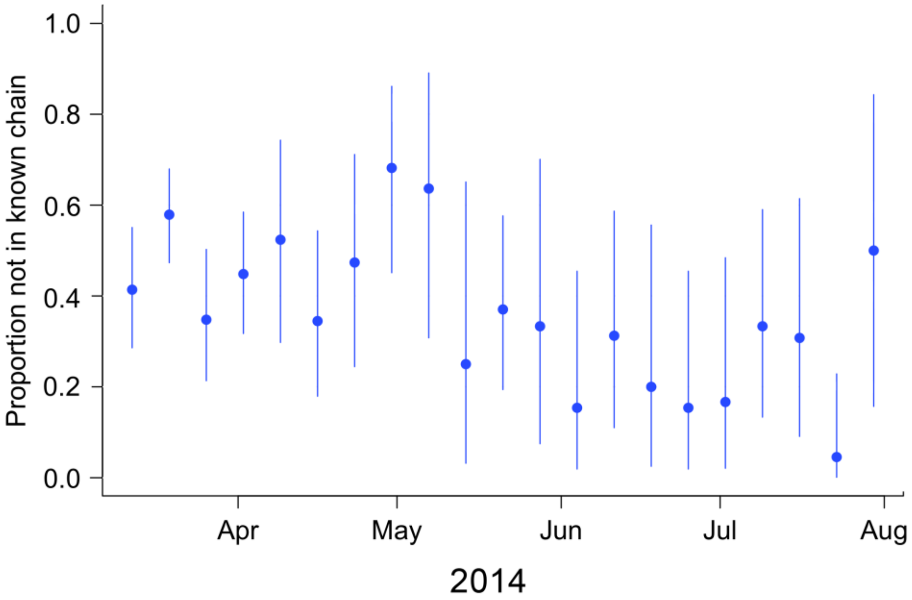

Technical Appendix Figure 2. Proportion of weekly new cases of Ebola virus disease in Guinea in 2015 that were not a known contact of an existing case, and were not part of an existing transmission chain. Points show expected proportion, and lines show 95% binomial CIs. Data were obtained from the World Health Organization (*16*).

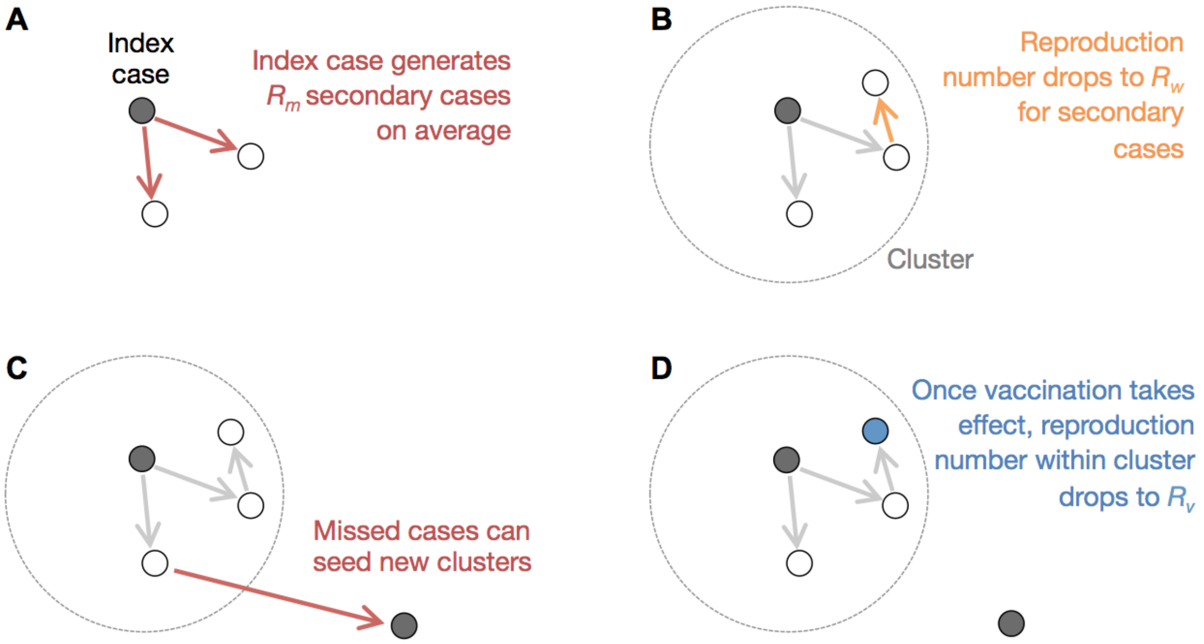

Technical Appendix Figure 3. Model schematic for effectiveness of ring vaccination as control strategy for Ebola virus disease. A) Index cases generate an average of R_m secondary cases. B) Secondary cases within cluster have a lower reproduction number (R_w). C) There is a probability ρ that a secondary case will be missed and go on to seed a new transmission cluster. D) Once vaccination takes effect, the reproduction number for cases within the cluster decreases to R_v.

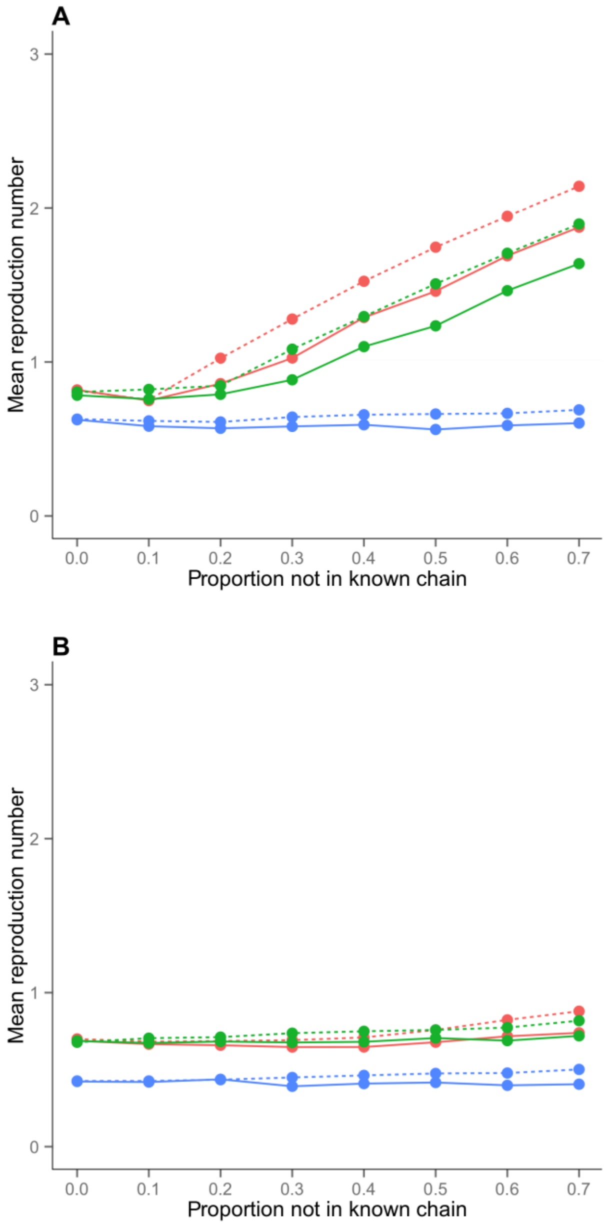

Technical Appendix Figure 4. Overall reproduction number in different scenarios for effectiveness of ring vaccination as control strategy for Ebola virus disease. A) Mean overall reproduction number across 1,000 simulated outbreaks in the Conakry, Guinea, transmission scenario. Red lines indicate no vaccination, green lines indicate ring vaccination, blue lines indicate mass vaccination, solid lines indicate outbreaks that started with 1 index case, and dashed lines indicate outbreaks that started with 5 index cases. Vaccine has 80% efficacy in the model. B) Mean reproduction number in the partial control scenario.

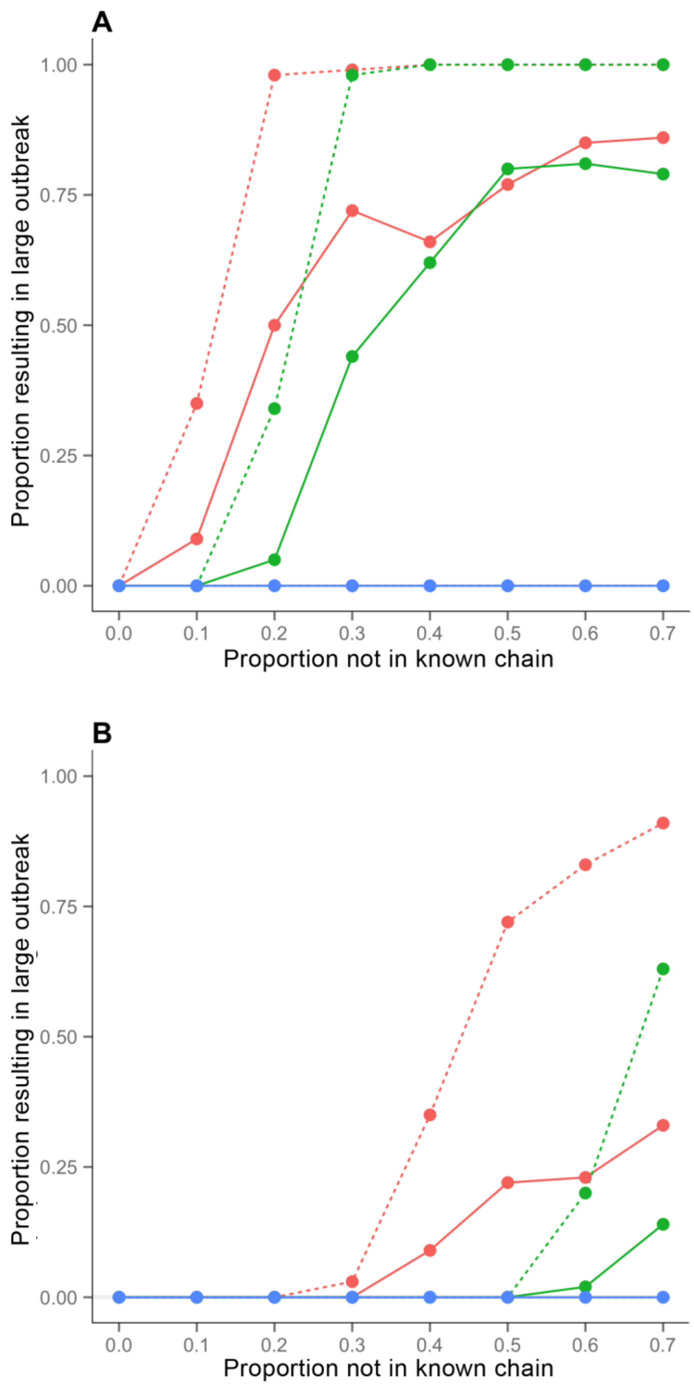

Technical Appendix Figure 5. Effectiveness of vaccination strategies under different transmission scenarios, when vaccine has 95% efficacy, as control strategy for Ebola virus disease. A) Proportion of simulations that led to a large outbreak (defined as >500 clusters) in the Conakry, Guinea, transmission scenario. Red lines indicate no vaccination, green lines indicate ring vaccination, blue lines indicate mass vaccination, solid lines indicate outbreaks that started with 1 index case, and dashed lines indicate outbreaks that started with 5 index cases. We simulated 1,000 outbreaks and calculated the proportion

that resulted in >500 clusters. When the space between the red and green lines is large, it suggests that ring vaccination would provide substantial additional value over standard public health control measures alone. B) Proportion of simulations that led to a large outbreak in the partial control scenario.

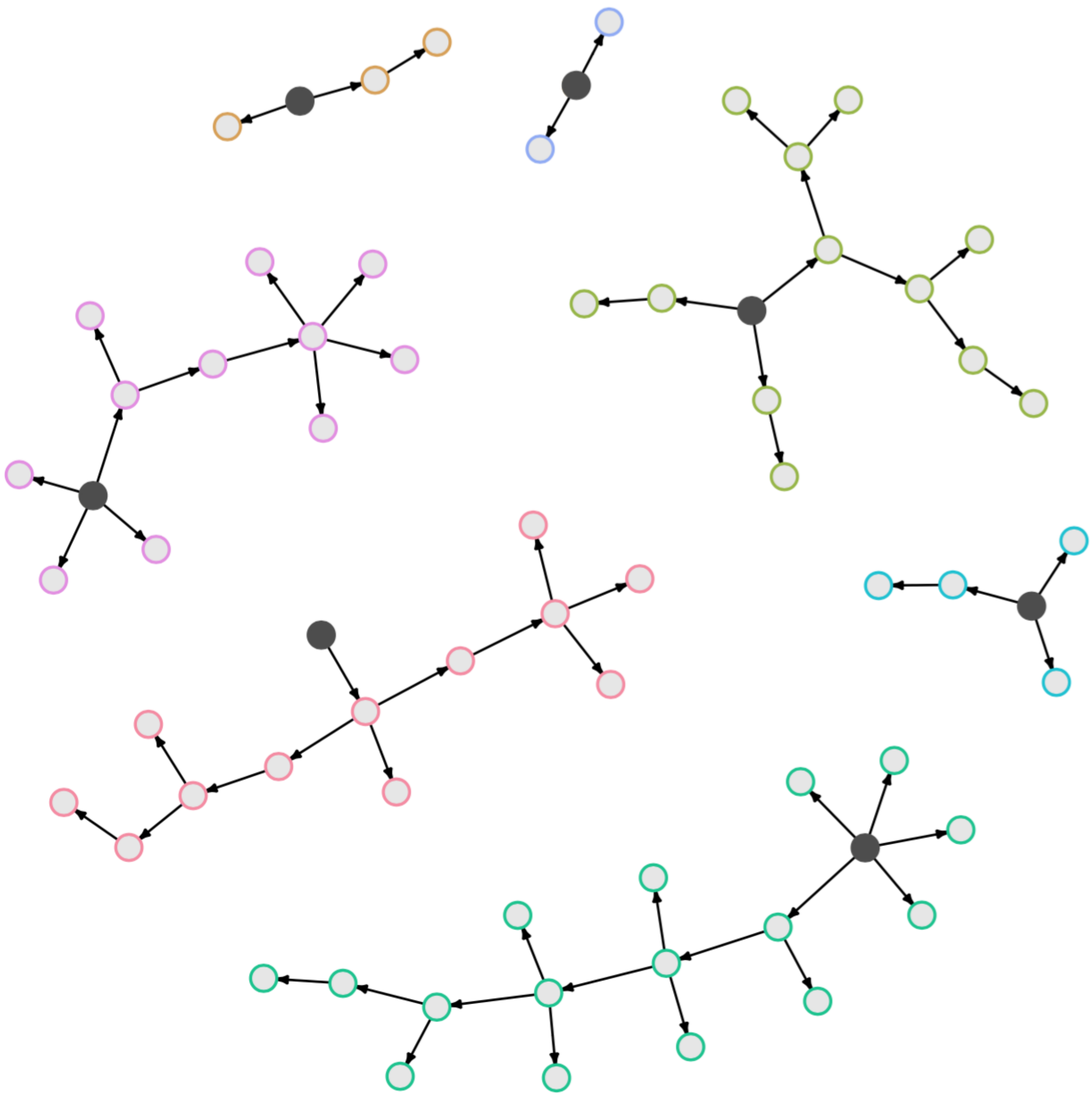

Technical Appendix Figure 6. Chains of transmission of Ebola virus disease generated in a simulated outbreak starting with 2 infected persons under the partial control scenario. Black points indicate the index case within each cluster, and arrows indicate routes of transmission. Within each cluster, we assumed there was a 15% probability that a secondary case would not be included in the existing chain, and would instead seed a new cluster (these missed links are not shown).
